# Identification of potential inhibitor targeting enoyl-acyl carrier protein reductase (InhA) in *Mycobacterium tuberculosis*: a computational approach

**DOI:** 10.1007/s13205-013-0146-0

**Published:** 2013-06-18

**Authors:** V. Shanthi, K. Ramanathan

**Affiliations:** 1 Industrial Biotechnology Division, School of Bio Sciences and Technology, VIT University, Vellore, 632014 Tamil Nadu India; 2 Bioinformatics Division, School of Bio Sciences and Technology, VIT University, Vellore, 632014 Tamil Nadu India

**Keywords:** INH-NAD adduct, Virtual screening, Molecular docking, Bioavailability, Normal mode analysis

## Abstract

The explosive global spreading of multidrug resistant *Mycobacterium tuberculosis* (*Mtb*) has provoked an urgent need to discover novel anti-TB agents. Enoyl-acyl carrier protein reductase from *Mtb* is a well-known and thoroughly studied target for anti-tuberculosis therapy. In the present analysis, virtual screening techniques performed from Drug bank database by utilizing INH-NAD adduct as query for the discovery of potent anti-TB agents. About 100 molecules sharing similar scaffold with INH-NAD adduct were analyzed for their binding effectiveness. The initial screening based on number of rotatable bonds gave 42 hit molecules. Subsequently, physiochemical properties such as toxicity, solubility, drug-likeness and drug score were analyzed for the filtered set of compounds. Final data reduction was performed by means of molecular docking and normal mode docking analysis. The result indicates that DB04362, adenosine diphosphate 5-(beta-ethyl)-4-methyl-thiazole-2-carboxylic acid could be a promising lead compound and be effective in treating sensitive as well as drug-resistant strains of *Mtb*. We believe that this novel scaffolds might be the good starting point for lead compounds and certainly aid the experimental designing of anti-tuberculosis drug in a short time.

## Introduction

Tuberculosis (TB) is a chronic infectious disease caused by mycobacteria such as *Mycobacterium bovis*, *Mycobacterium africanum* and mainly *Mycobacterium tuberculosis* (Aziz et al. 2006). One-third of the world’s population is infected with *Mycobacterium tuberculosis*, the etiological agent of TB, resulting in 9.2 million new cases and 1.7 million deaths in 2006 (Floyd and Pantoja 2008). Globally in 2007, there were a predictable 13.7 million chronic active cases. In 2010, there were 8.8 million new cases and 1.5 million associated deaths occurred in developing countries. Active TB is usually treated with isoniazid (INH) in association with one or more other anti-TB drugs but multidrug-resistant TB (MDR-TB) and very recently extensively drug-resistant TB (XDR-TB) have become a serious and unsolved public health problem (Aziz et al. 2006; Pasqualoto et al. 2004; Wei et al. 2003; Morlock et al. 2003; Ormerod 2005). INH is a prodrug and must be activated by the catalase–peroxidase KatG (Zhang et al. 1992; Johnsson and Schultz 1994; Marcinkeviciene et al. 1995^;^ Johnsson et al. 1997; Wang et al. 1998) and the isoniazid-activated intermediate forms an isonicotinoyl-NAD adduct (INH-NAD), through addition of either an isonicotinic acyl anion to NAD^+^ or an isonicotinic acyl radical to an NAD· radical (Rozwarski et al. 1998). After activation, it inhibits enoyl-acyl carrier protein reductase (InhA). Inhibition of this activity by INH blocks the biosynthesis of mycolic acids, which are major lipids of the mycobacterial envelope (Quemard et al. 1996; Marrakchi et al. 2000). INH-NAD adduct is a slow tight binding competitive inhibitor of InhA that binds with an overall dissociation constant of 0.75 nM (Rawat et al. 2003). Missense mutations in the inhA structural gene have been identified in clinical isolates of *Mycobacterium tuberculosis* resistant to INH. More studies have also demonstrated that the mutations within katG in *Mtb* are common in INH-resistant strains (Heym et al. 1995). The mutations within inhA have been reported up to 32 % in INH resistant strains (Telenti et al. 1997; Morris et al. 1995; Lee et al. 1999; Kiepiela et al. 2000). Mutations in katG and inhA account for up to 80 % of INH-resistant strains (Morris et al. 1995; Musser et al. 1996), whereas the mutations in katG alone account for the majority of INH resistant strains (Cynamon et al. 1999; Torres et al. 2000). This indicates that high prevalence of resistance to INH was observed, mainly due to emerging KatG mutants that do not activate or poorly activate INH. Therefore, it has been suggested that compounds that inhibit the ultimate target of INH but do not require activation by KatG have tremendous promise as novel drugs for combating MDR-TB and XDR-TB (Rawat et al. 2003; Basso et al. 1998). Keeping in view, the importance of enoyl-acyl carrier protein reductase (InhA), an enzyme involved in the biosynthesis of mycolic acids and low outcome of inhibitors using experimental procedures, we have made an attempt to screen inhibitors of InhA by virtual screening procedures.

Virtual screening (VS) is a widely used method that has been shown to be successful in a variety of studies, although it also has many shortcomings (Oprea and Matter 2004; Chen 2008). In the past few years, many reports indicated that virtual screening techniques proved to be effective in making qualitative predictions that discriminated active from inactive compounds (Kitchen et al. 2004). The use of experimentally derived protein structures and a hybrid computational method that combines the advantages of docking algorithms with dynamic structural information provided by normal mode analysis certainly provide improved library enrichments virtual screening process. Furthermore, this is the first report of virtual screening for InhA inhibitors and the results could aid experimental studies and the rational development of novel drugs against *Mtb*.

## Materials and methods

### Data set preparation

The native and mutant (I21V) type’s coordinates of INH-NAD adduct complexed to InhA were taken from the Brookhaven Protein Data Bank (PDB) (Berman et al. 2000). The corresponding PDB codes were 2IDZ and 2IE0, respectively. The adduct was extracted from the PubChem, a database maintained in NCBI (Wishart et al. 2008; Feldman et al. 2006) and SMILES strings was collected and submitted to CORINA for constructing the 3D structure of the INH-NAD adduct (Gasteiger et al. 1990). All the water molecules and the hetero atoms were removed. Energy minimization for native and mutant proteins was carried out using GROMACS package 4.5.3 (Hess et al. 2008; Spoel et al. 2005) adopting the GROMOS43a1 force field parameters before performing molecular docking experiment. The screening performed with the aid of PubChem and Drug bank database.

### Virtual screening

VS (Shoichet 2004) is the computational analogue of biological screening. The approach has become increasingly popular in the pharmaceutical research for lead identification. The basic goal of the VS is the reduction of the massive virtual chemical space of small organic molecules, to screen against a specific target protein, to a manageable number of the compounds that inhibit a highest chance to lead to a drug candidate. Two different databases such as PubChem and Drug bank were used for searching new lead compounds by employing the INH-NAD adduct as query (Bolton et al. 2011; Wishart et al. 2008). The numbers of molecules in each of the database are 85 million and 140,000, respectively. Screening was carried out by restricting the number of rotatable bonds to a maximum of 12 (Muegge 2003; Oprea 2000). Several hits were obtained from each of the databases, which were further screened using molecular docking studies. The SMILES strings were used for constructing three-dimensional structures of lead compounds.

### Identification of binding site residues for enoyl-acyl carrier protein reductase

It was a challenging task to extrapolate a mechanism of action from the view of 3D structures. Detailed biochemical information about the enzyme can be used to design substrate or transition state analogues, which can then be bound into the enzyme for structure determination. These can reveal binding site locations and identify residues, which are likely to take part in the receptor–ligand interaction. From this, a catalytic mechanism can be proposed. In order to identify the binding residues in the structure of enoyl-acyl carrier protein reductase, we submitted the native and mutant complex structure (PDB ID: 2IDZ and 2IE0) into the ligand contact tool (LCT) program (Lopez et al. 2007). This program calculates contacts between the binding residues of enoyl-acyl carrier protein reductase receptor with INHNAD using default parameters.

### Molecular docking

Docking was performed with the help of the PatchDock (Duhovny et al. 2005). It is a geometry-based molecular docking algorithm. The PatchDock algorithm divides the Connolly dot surface representation (Connolly 1983a, b) of the molecules into concave, convex and flat patches. Then, complementary patches are matched in order to generate candidate transformations. Each candidate transformation is further evaluated by a scoring function that considers both geometric fit and atomic desolvation energy (Zhang et al. 1997). Finally, root mean square deviation (RMSD) clustering is applied to the candidate solutions to discard redundant solutions. The input parameters for the docking are the PDB coordinate file of the protein and ligand molecule. This algorithm has three major stages: (1) molecular shape representation, (2) surface patch matching, and (3) filtering and scoring. Furthermore, the steric clashes, introduced by PatchDock, algorithm are removed with the aid of FireDock algorithm (Andrusier et al. 2007; Mashiach et al. 2008). FireDock refines side chain positions and relative protein orientations. After steric clashes are removed, an energy-like function is used to rank the docking models. This interface energy score is a weighted combination of softened van der Waals, desolvation, electrostatics, hydrogen bonding, disulfide bonding, π-stacking, aliphatic interactions, and rotamer preferences (Andrusier et al. 2007); docking results were screened by means of FireDock algorithm.

### Prediction of physiochemical properties

Successful drug discovery requires high-quality lead structures which may need to be more drug-like than commonly accepted (Proudfoot 2002). The hits were screened using drug-likeness, drug score and toxicity characteristics. These physicochemical properties were therefore calculated for the filtered set of hits using the program OSIRIS (Sander 2001). The OSIRIS program calculates the drug-likeness based on a list of about 5,300 distinct sub-structure fragments created by 3,300 traded drugs as well as 15,000 commercially available chemicals yielding a complete list of all available fragments with associated drug-likeness. The drug score combines drug-likeness, cLogP, logS, molecular weight, and toxicity risks as a total value which may be used to judge the compound’s overall potential to qualify for a drug.

### Normal mode analysis

The exploration of molecular motions of biological molecules and their assemblies by simulation approaches such as molecular dynamics has provided significant insights into structure–function relationships in small biological systems. Normal mode analysis (NMA) provides an alternative to molecular dynamics for studying the motions of macromolecules. The time scale accessible to theoretical work is extended with normal mode analysis, and this approach has been proven extremely useful for studying collective motions of biological systems. (Noguti and Nishikawa 1983; Levitt et al. 1985) Normal mode analysis is a powerful tool for predicting the possible movements of a given macromolecule. It has been shown recently that half of the known protein movements can be modeled by using at most two low-frequency normal modes (Tama and Sanejouand 2001). Applications of NMA cover wide areas of structural biology, such as the study of protein conformational changes upon ligand binding, membrane channel opening and closure, potential movements of the ribosome, and viral capsid maturation. Another newly emerging field of NMA is related to protein structure determination by X-ray crystallography, where normal mode perturbed models are used as templates for diffraction data phasing through molecular replacement. Elnemo is a web interface to the elastic network model that provides a fast and simple tool to compute, visualize and analyze low-frequency normal modes of large macro-molecules and to generate a large number of different starting models for use in molecular replacement (Suhre and Sanejouand 2004). Using this interface, each docked complex was analyzed with default parameters to investigate the active site residues by normal mode analysis.

## Results and discussion

### Virtual screening

Virtual screening is the computational analogue of biological screening. It uses computer-based methods to discover new ligands on the basis of biological structures. This technique mainly focuses on comparing molecular similarity analyses of compounds with known and unknown moiety. Here, we have performed the virtual screening analysis by using INH-NAD adduct, an active intermediate molecule. The result indicates that 332 hits from PubChem and 100 hits from Drug Bank were identified similar to the INH-NAD adduct. Successful drug discovery requires high-quality lead structures which may need to be more drug-like than is commonly accepted (Proudfoot 2002). The initial screening was carried out by restricting the number of rotatable bonds to a maximum of 12, which reduced the number of hits to 42 from drug bank whereas PubChem database did not show the reasonable hit compounds. The results are shown in Table 1. Therefore, the subsequent analysis was carried out with the aid of 42 molecules screened from drug bank database.

**Table 1:** Number of rotatable bonds obtained from the Molinspiration program

S. no.	Compound ID	Number of rotatable bonds	S. no.	Compound ID	Number of rotatable bonds
1	DB00157	11	22	DB01860	8
2	DB01907	11	23	DB03909	8
3	DB03797	11	24	DB02661	6
4	DB02498	11	25	DB04366	6
5	DB01893	9	26	DB04362	10
6	DB04497	11	27	DB02930	8
7	DB02059	9	28	DB03070	11
8	DB01842	8	29	DB01812	6
9	DB04099	11	30	DB03230	7
10	DB03431	6	31	DB02902	8
11	DB02363	8	32	DB02527	1
12	DB03363	11	33	DB03732	11
13	DB03969	11	34	DB00131	4
14	DB03478	11	35	DB04395	8
15	DB03020	11	36	DB02098	6
16	DB00171	8	37	DB02483	9
17	DB03893	11	38	DB03708	6
18	DB01660	8	39	DB04418	9
19	DB04554	6	40	DB03222	8
20	DB01774	9	41	DB02694	9
21	DB04071	11	42	DB07205	8

### Binding site residues analysis

**Fig. 1:**
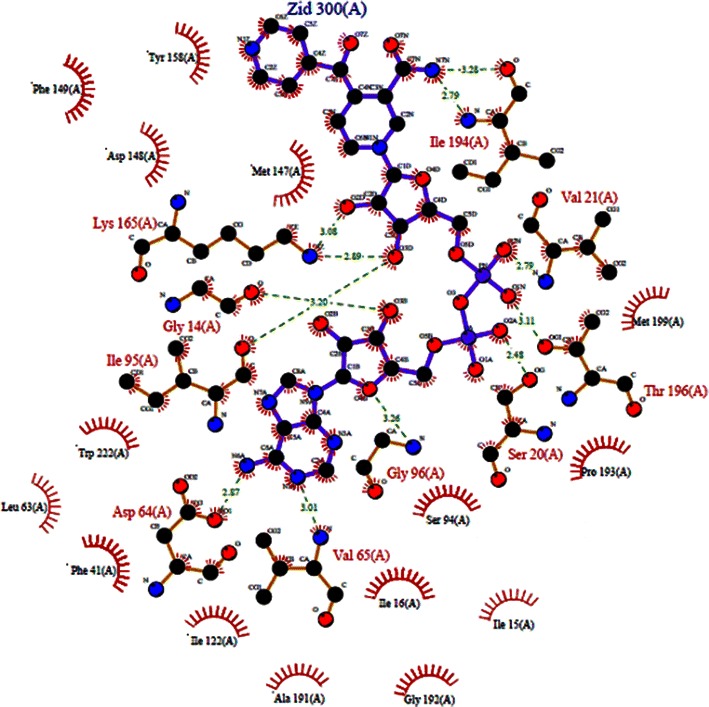
INH-NAD adduct bound with enoyl-acyl carrier protein reductase. The figure was rendered using the program LIGPLOT

The binding site residues in the structure of enoyl-acyl carrier protein reductase were obtained from LCT program by using the complex structure of enoyl-acyl carrier protein reductase bound with INH-NAD adduct (PDB ID: 2IE0). The results indicate for 2IE0 are a total of 10 amino acid residues, viz, G-14, S-20, V-21, D-64, V-65, I-95, G-96, K-165, I-194 and T-196. The LIGPLOT (Wallace et al. 1995) tool was used to illustrate the contacts between mutant protein binding residues and INH-NAD adduct shown in Fig. 1.

### Docking studies of mutant enoyl-acyl carrier protein reductase with inhibitor

**Fig. 2:**
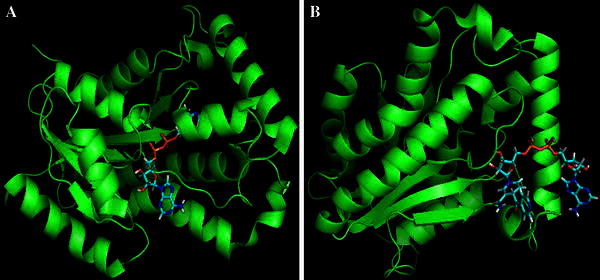
Docked complex of INH-NAD adduct with native (**a**) and mutant (**b**) type enoyl-acyl carrier protein reductase

**Fig. 3:**
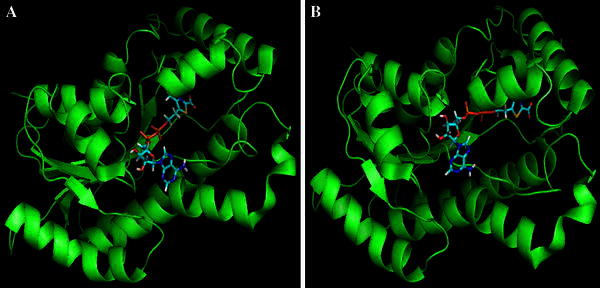
Docked complex of DB04362 with native (**a**) and mutant (**b**) type enoyl-acyl carrier protein reductase

In order to gain insight into possible binding modes of the inhibitor, INH-NAD adduct, was docked into the ligand-binding domain of the native and mutant type of InhA using the program PatchDock. It is well known that the scores calculated by docking programs do not usually permit the exact reproduction of the binding mode of assayed compounds. Hence, we have further screened the PatchDock results with the aid of FireDock program. This will be of immense importance in obtaining the binding affinity of INH-NAD adducts with the target structures. The binding affinity between INH-NAD adducts and the target structures determined by fireDock program were −43.70 and −48.27 kcal/mol for the native and mutant structures, respectively. This clearly indicates that mutation at the position I21V in the target structure leads resistance to drug molecule, isoniazid, but not to the INH-NAD adduct. Subsequently, all 42 selected hits were docked into the InhA active site in the same way in order to understand the binding affinity of the lead compounds against the native and mutant type proteins. We understand that 9 hits showed greater binding affinity with the mutant type (I21V) compared to INH-NAD adduct. The result was shown in Table 2. In particular, five compounds such as DB04362, DB03893, DB00157, DB02498 and DB04418 showed greater binding affinity with both native and mutant type structure. The compound DB04362 showed greatest binding affinity than other lead compounds considered in our study. The docked complex structure of INH-NAD adduct and DB04362 with native and mutant type structure shown in Figs. 2 and 3.

**Table 2:** Binding free energy analysis of lead compounds

S. no.	Compound	Binding free energy with native *InhA* (kcal/mol)	Binding free energy with mutant *InhA* (kcal/mol)
1	INH-NAD	−43.70	−48.27
2	DB04362	−55.61	−57.27
3	DB02483	−38.69	−56.31
4	DB03893	−45.89	−54.34
5	DB04497	−24.28	−54.03
6	DB03732	−43.04	−53.45
7	DB03478	−38.92	−53.43
8	DB00157	−56.11	−53.35
9	DB02498	−46.73	−51.31
10	DB04418	−54.80	−50.58

### In silico toxicities, solubility, drug-likeness and drug score profiles

Many drug candidates fail in the clinical trials, reasons are unrelated in the potency against the intended drug target. Pharmacokinetic and toxicity issues are blamed for more than half of all failure in the clinical trials. Therefore, it is essential to evaluates Pharmacokinetic and toxicity of small molecules. Drug solubility (log S) is an important factor that affects the movement of a drug from the site of administration into the blood. It is known that insufficient solubility of drug can lead to poor absorption (Muegge 2003). Our estimated log S value is a unit stripped logarithm (base 10) of a compound’s solubility measured in mol/liter. There are more than 80 % of the drugs on the market that have an (estimated) log S value greater than −4. Table 3 shows solubility of the seven compounds that were found in the comparable zone with that of standard drugs to fulfill the requirements of solubility and could be considered as a candidate drug for oral absorption.

**Table 3:** Physicochemical Properties of lead compounds

S. no.	Compound	Toxicity risks				Osiris calculations		
		Mutagenic	Tumorigenic	Irritant	Reproductive effective	Log S	DL	DS
1	INH-NAD	–	–	–	–	−5.75	−16.27	0.17
2	DB04362	–	–	–	–	−2.11	−12.8	0.3
3	DB02483	–	–	–	–	−3.1	−13.3	0.3
4	DB04497	–	–	–	–	−2.43	−15.54	0.28
5	DB03732	–	–	–	–	−2.44	−13.48	0.27
6	DB03478	–	–	–	–	−3.32	−15.52	0.29
7	DB00157	–	–	–	–	−4	−15.43	0.25
8	DB02498	–	–	–	–	−3.67	−13.78	0.25

### Drug-likeness

Currently, there are many approaches to assess a compound drug-likeness based on topological descriptors, fingerprints of molecular drug-likeness structure keys or other properties such as C log P and molecular weight. In this work, Osiris program (Sander 2001) was used for calculating the fragment-based drug-likeness of the most active compounds and comparing them with INH-NAD adduct. Seven compounds showed a little improvement of drug-likeness values than INH-NAD adduct and it is shown in Table 3. The drug scores of the potent compounds have also been determined in the present study.

### Drug score

We have calculated overall drug score (DS) for the lead compounds as compared with that of standard drugs INH-NAD adduct. The drug score combines drug-likeness, miLogP, log S, molecular weight and toxicity risks in one handy value that may be used to judge the compound’s overall potential to qualify for a drug. The result is shown in Table 3. The reported lead compounds showed moderate to good drug score as compared with standard drug used. The drug score of the seven compounds showed a good score which is of significantly higher value than that of the INH-NAD adduct.

The toxicity risk predictor locates fragments within a molecule, which indicate a potential toxicity risk. Toxicity risk alerts are an indication that the drawn structure may be harmful concerning the risk category specified. Data evaluated in Table 3 indicate that all the seven lead compounds were supposed to be non-mutagenic, non-irritating with no tumorigenic effects when run through the mutagenicity assessment system comparable with standard drugs used.

### Normal mode docking analysis

**Fig. 4:**
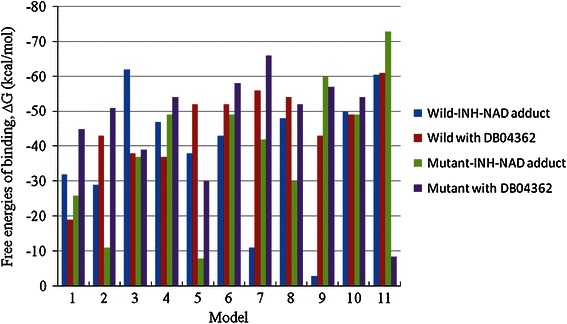
Comparison of free energies of binding for the INH-NAD adduct with native (*black*) and mutant (*red*) structures using normal mode analysis

It has been recently shown that half of the known protein movements can be modeled by using at most too low-frequency normal modes for explaining collective large amplitude motions of proteins in different conformational states (Delarue and Dumas 2004). These motions typically describe conformational changes which are essential for the functioning of proteins (Alexandrov et al. 2005). Hence, the lowest frequency mode (mode 7) (Choudhury et al. 2010) was used for our docking study. The normal mode analysis generates 11 possible confirmations between DQMIN of −100 and DQMAX of 100 with DQSTEP step size of 20 (Suhre and Sanejouand 2004). It is to be noted that understanding the binding affinity between the target and the drug based on relevant normal modes will authorize the strength of docking process (Cavasotto et al. 2005). Hence, entire trajectory files from the lowest frequency mode were used as the input for docking analysis. Each harmonic vibrational mode derived from NMA simulates a state of the system in which all particles are oscillating with the same characteristic frequency and, therefore, the method is often referred to as collective motion analysis. Unfortunately, the protein structure deposited in the PDB corresponds to single conformation. Therefore, NMA, particularly with a simple elastic network model, can be helpful for simulation of an active site motion. The three-dimensional structure of INH-NAD adduct was generated by using the tool CORINA (Gasteiger et al. 1990). The normal mode-based docking result is shown in Fig. 4. We observed that free energy of binding for the lead compound, DB04362, was significantly higher than INH-NAD adduct in all the 11 conformations generated by means of normal mode analysis. This clearly indicates the effective binding of DB04362 than INH-NAD adduct with native and mutant structure of InhA.

## Conclusion

Despite the availability of effective treatments, tuberculosis still persists as epidemiological entities that cause chronic, crippling illness and death on a large scale. The main reason for this is the emergence of drug-resistant strains due to poor compliance among the patients to their lengthy treatment regimens. Therefore, in the present study, we identified novel drugs that are active against the drug-resistant as well as non-resistant strains, and can shorten their treatment durations using virtual screening protocols. Initially, virtual compounds were subjected to molinspiration program and screening was carried out by restricting the number of rotatable bonds to a maximum of 12. Subsequently the screened molecules were docked at the active site of *Mtb* InhA to select inhibitors establishing favorable interactions. Finally, toxicity and drug-likeness were evaluated in order to screen the high-quality lead structure. Several potential drug-like inhibitors have been screened out showing strong binding affinity to *Mtb* InhA. Furthermore, normal mode analysis indicates that the compound, adenosine diphosphate 5-(beta-ethyl)-4-methyl-thiazole-2-carboxylic acid (DB04362) displayed strong binding affinity with both the native and mutant type InhA. Hence, we believed that DB04362 represents promising starting point as a lead compound for *Mtb*. Though experimental studies are indispensable to mark them as lead compound for the development of novel drugs against *Mtb*, however, screened out inhibitors would undoubtedly aid the experimental designing of anti-tubercular agents expeditiously.
